# Walking Ability Is a Major Contributor to Fear of Falling in People with Parkinson's Disease: Implications for Rehabilitation

**DOI:** 10.1155/2012/713236

**Published:** 2011-09-19

**Authors:** Maria H. Nilsson, Gun-Marie Hariz, Susanne Iwarsson, Peter Hagell

**Affiliations:** ^1^Department of Health Sciences, Lund University, Box 157, SE-221 00 Lund, Sweden; ^2^Department of Community Medicine and Rehabilitation, Umeå University, SE-901 87, Umeå, Sweden; ^3^Department of Pharmacology and Clinical Neuroscience, Umeå University, SE-901 87, Umeå, Sweden; ^4^School of Health and Society, Kristianstad University, SE-291 88 Kristianstad, Sweden

## Abstract

Although fear of falling (FOF) is common in people with Parkinson's disease (PD), there is a lack of research investigating potential predictors of FOF. This study explored the impact of motor, nonmotor, and demographic factors as well as complications of drug therapy on FOF among people with PD. Postal survey data (including the Falls Efficacy Scale, FES) from 154 nondemented people with PD were analyzed using multiple regression analyses. Five significant independent variables were identified explaining 74% of the variance in FES scores. The strongest contributing factor to FOF was walking difficulties (explaining 68%), followed by fatigue, turning hesitations, need for help in daily activities, and motor fluctuations. Exploring specific aspects of walking identified three significant variables explaining 59% of FOF: balance problems, limited ability to climb stairs, and turning hesitations. These results have implications for rehabilitation clinicians and suggest that walking ability is the primary target in order to reduce FOF. Specifically, balance, climbing stairs, and turning seem to be of particular importance.

## 1. Introduction

People with Parkinson's disease (PD) have an increased risk of falling [1], and fear of falling (FOF) is also more common and pronounced compared to controls [2–6]. FOF has been described as an ongoing concern about falling, a loss of balance confidence, low fall-related self-efficacy, or as activity avoidance [7–11]. 

The prevalence of FOF in people with PD has been reported to range from 35% to 59% [2, 12–16], although a study that included only men reported a lower prevalence (18%) [17]. It is even more common and pronounced among fallers [2, 6, 12–14, 17–19]. FOF can cause social isolation [20], and up to 70% of people with PD report activity limitations due to FOF [2, 21]. It is thus important for rehabilitation clinicians to understand the factors contributing to FOF. 

Successful interventions need to be based on an understanding of factors associated with (and potentially influencing) the target of the intervention. That is, if rehabilitation aims to reduce FOF, it should target factors that may influence FOF. With respect to FOF in PD, weak to moderate associations (Spearman correlations (*r*_*s*_)) have been found between FOF and age (*r*_*s*_, ≤ 0.08), PD duration (*r*_*s*_, <0.29), and disease severity (*r*_*s*_, 0.47) [14, 22]. Previous studies have also shown that FOF relates to freezing of gait (FOG) [15, 23], physical functioning [14], gait tests [5, 14, 22, 24], balance [3, 14, 22], mobility, activities of daily living (ADL) [14, 21], and sex [14]. However, those studies have relied on bivariate analyses, and none has simultaneously taken a broader range of independent variables (e.g., motor symptoms, drug therapy complications such as motor fluctuations and dyskinesias, nonmotor symptoms, and demographic factors) into account. The objective of this study was to explore the potential contributions of motor, nonmotor, and demographic factors, as well as complications of drug therapy, on FOF among people with PD.

## 2. Participants and Methods

Data were collected by a postal survey to a sample of people with idiopathic PD [25]. All individuals with PD that received care at a Swedish university hospital were considered for inclusion in the study. Exclusion criteria constituted dementia or severe cognitive impairment as determined by their respective PD-specialized nurse clinicians. The survey was sent to 282 individuals (39% women) followed by a reminder about ten days later. Of 231 returned questionnaires, 38 were returned blank and two were returned to sender due to a change of address. There were thus 191 survey respondents (43% women; conservative total response rate, 68%). Six of these had left the included FOF-questionnaire completely blank, and total scores could not be computed for another 31 participants due to missing data. These 37 persons were excluded from the analysis. Excluded participants did not differ (*P* ≥ 0.153) from those included with respect to sex, age, and PD duration. Characteristics of the final study sample (*n* = 154) are presented in Table 1. The investigators did not have access to patient details (beyond those provided by survey responders) or addresses. The study was conducted in accordance with the Declaration of Helsinki, and all participants gave their informed consent.

### 2.1. Survey Questions and Instruments

In addition to demographic questions, the survey included a set of questions on the presence or absence (no/yes) of motor fluctuations (i.e., a fluctuating effect of anti-PD medications with periods of more severe motor symptoms), dyskinesias (i.e., involuntary, irregular, twisting, and/or jerky movements), comorbidity, FOF, falls during the past six months (described and defined as by Lamb et al. [26]), near falls (described and defined as by Gray and Hildebrand [27]), and need of help from others in daily activities. Overall perceived PD severity was self-rated as “mild,” “moderate,” or “severe.” In addition, participants were asked whether they had responded to the survey themselves (with or without assistance in reading and/or writing).

A battery of self-administered questionnaires was included. The Falls Efficacy Scale (FES) conceptualizes FOF as low fall-related self-efficacy [8]. The Swedish version, FES(S), includes 13 items (activities) rated from 0 (not confident at all) to 10 (completely confident) [14, 28]. The maximum total score is 130 points, and a higher score denotes “better” balance confidence. The Functional Assessment of Chronic Illness Therapy-Fatigue scale (FACIT-F) consists of 13 items with a total score ranging between 0–52 (higher scores = less fatigue) [29, 30]. The physical functioning (PF) scale from the Short Form-36 (SF-36) includes ten items, and the total score can range between 0–100 (higher scores = better) [31, 32]. The self-administered version of the FOG Questionnaire (FOGQsa) consists of six items graded 0–4 (higher = worse) [15]. In this study we only used items 3 (freezing: “*Do you feel that your feet get glued to the floor while walking, making a turn or when trying to initiate walking (freezing)?”*) and 6 (turning hesitations: “*During the past week, how long have your typical “freezing” episodes been when making a turn?”*) of the FOGQsa. Those scoring ≥1 on item 3 were categorized as “freezers,” and those scoring ≥1 on item 6 were considered to have turning hesitations. The generic version of the Walk-12 (Walk-12G) assesses walking difficulties in everyday life from the individual's perspective [33–35]. The total Walk-12G score ranges between 0–42 points (higher scores = worse). In this study, item 6 (*“Have you had problems balancing when standing or walking?*”) of the Walk-12G (graded 0–4) was specifically used to identify and describe balance problems. Those scoring ≥1 were considered having balance problems. The pain section of the Nottingham Health Profile (NHP-Pain) has eight items and yields a total score between 0–100 (higher scores = more pain) [36, 37].

All included patient-reported rating scales have previously been found to have acceptable validity and reliability in people with PD [14, 15, 30, 32, 35, 37]. Reliabilities (coefficient alpha) in this study were as follows: FES(S), 0.98; FACIT-F, 0.85; PF, 0.93; Walk-12G, 0.96; NHP-Pain, 0.85. Corrected item-total correlations in this study were all ≥0.30. These data support the adequacies of scores used in this study [38].

### 2.2. Statistical Analysis

Data were checked regarding underlying assumptions and described and analyzed accordingly using PASW version 18 (SPSS Inc., Chicago, IL). The alpha level of significance was set at 0.05 (2-tailed, exact *P*-values were used).

Spearman correlations (*r*_*s*_) and Mann-Whitney *U*-tests were used for bivariate analyses of associations with FOF, that is, FES(S). Variables significantly associated with FES(S) scores in bivariate analyses were then entered as independent variables in regression models with FES(S) scores as the dependent variable. To ease interpretation, all scores were adjusted to be in the same direction (higher scores = more problems) before being entered into the regression analyses. 

A first regression model (method: forward) included motor, nonmotor, and demographic factors as well as drug therapy complications (i.e., fluctuations and dyskinesias) as independent variables. Further details about the included independent variables are provided as footnotes in Table 2. Based on results from the first model, a second model was explored (method: enter with manual backward deletion) consisting of items from scales found significant in the first model. These items (independent variables) were selected based on whether they appeared to represent specific aspects potentially suitable for rehabilitation interventions, in combination with clinical considerations. Details about the included independent variables are provided as footnotes in Table 3.

## 3. Results

Eighty-five % (131/154) of the participants responded completely independently to the postal survey, whereas the rest attained assistance in reading or writing. The included 154 participants had a median FES(S) score of 114 (q1–q3, 69–130; min-max, 0–130) and 29% scored at maximum, that is, 130. According to the dichotomous FOF-question, 45% (67 out of 149) perceived themselves as having FOF. In addition, 76% (112/148) of the participants experienced balance problems when standing or walking. Perceived PD severity was rated as “moderate” by 96 participants and ranged from “mild” (*n* = 43) to “severe” (*n* = 14). 

Bivariate analyses are presented in Table 1. FES(S) scores demonstrated the weakest correlation with age (*r_s_*, −0.24) and the strongest (*r*_*s*_, −0.82) with walking difficulties. Those reporting the presence of motor fluctuations and dyskinesias had significantly (*P* ≤ 0.010) lower FES scores (i.e., more FOF) than those who did not (Table 1). Needing help from others in daily activities and experiencing FOG, turning hesitations, prior falls or near falls were also associated with more (*P* < 0.001) FOF (Table 1). 

In the first regression model, there were signs of multicollinearity between PF and Walk-12G scores (data not shown). PF was therefore omitted from the model in favor of the Walk-12G. This was done because the Walk-12G represents a more specific variable and exhibited a somewhat better reliability than the PF (0.96 versus 0.93). This resulted in a model with five significant independent variables, explaining 74% of the variance in FES(S) scores (Table 2). The strongest independent variable (as assessed by the standardized regression coefficients, *β*) was walking difficulties, which alone explained 68% of the variance in FES(S) scores. This was followed by fatigue, turning hesitations, needing help from others in daily activities, and motor fluctuations (Table 2). 

In the second explorative regression model, specific gait and balance items were entered as independent variables (Table 3). In this model, the original five response categories of Walk-12G items were recoded and entered as dummy variables: “not at all” (reference category), “a little,” and “moderately/quiete a bit/extremely,” that is, the three worst categories were merged into one (due to skewed response distributions). Two Walk-12G items were omitted from the model: item 11 (“Have you been limited in how far you are able to walk?”) due to signs of multicollinearity and item 12 (“Has your walking been slow?”) which was not significant. The final model included three significant independent variables explaining 59% of the variance in FES(S) scores (Table 3). The two strongest independent variables were (moderate to extreme) limitations in climbing stairs and balance problems. The third significant independent variable was turning hesitations.

## 4. Discussion

This study identified that walking disabilities contributed the strongest to FOF (i.e., low fall-related self-efficacy) in people with PD. That is, variations in self-rated walking ability could account for a high proportion (68%) of the variance in FES(S) scores. This is in line with previous studies showing a relationship between FOF and clinical gait tests [5, 14, 22, 24]. Furthermore, a mixed method pilot study found that FOF was universally reported in connection to everyday walking [39]. Our results have important implications for rehabilitation and suggest that walking difficulties should be the main target in order to reduce FOF. Arguably, such interventions may benefit from specifically targeting balance problems, stair climbing, and turning hesitations. These issues are of particular relevance for the physical therapist within the interdisciplinary team. 

The present finding of balance problems contributing independently to FOF is in accordance with previous results based on bivariate analyses [3, 14, 22]. It is noteworthy that prior falls or near falls were not independently associated to FOF when controlling for the other independent variables, despite the highly significant bivariate relationship demonstrated. This finding illustrates a major pitfall in relying on bivariate analyses. However, we did not register falls prospectively (as has been recommended [26]), and our sample had a relatively low proportion of fallers. Still, although further confirmatory studies are needed, our findings suggest that focus primarily should be put on perceived balance impairment rather than on fall prevention per se in order to reduce FOF.

Impaired balance is common among people with PD, which is confirmed by the fact that 76% of the participants in our study reported balance problems. This corresponds to the finding of Schrag et al., who reported that 65% of people with a PD duration of five years or more experience a postural instability [40]. Although gait and balance training are common in rehabilitation for people with PD, very few studies have investigated the effects on FOF. Some studies reported improvements after training [41–44], but it is unclear whether these were of clinical significance. In addition, none of these studies included a long-term followup and all used different outcome measures, which limit their comparability. Further studies are therefore warranted, which are of importance since pharmacological treatments have insufficient effects on gait and balance problems [45–47]. In addition, although deep brain stimulation in the subthalamic nuclei has been shown to positively influence FOF [48, 49], it is a surgical option only eligible for a minority of people with PD. 

We identified turning hesitations to be independently associated with FOF. While turning hesitations are related to FOG, it is noteworthy that freezing was not associated with FOF when controlling for the other independent variables in the identified model, despite the highly significant bivariate relationship demonstrated between FOF and FOG. This further illustrates the pitfall in relying on bivariate analyses. The present results suggest that turning hesitations should be more specifically addressed than FOG *per se* in order to reduce FOF. Turning is in fact impaired in mild PD [50], and rehabilitation clinicians (such as physical therapists) should therefore consider this already early on. Furthermore, moderate to extreme limitations in climbing stairs were also independently associated with FOF, and a previous study showed that stairs can cause considerable anxiety among people with PD [39]. This suggests that stair climbing should be considered more specifically both when assessing and treating people with PD. 

In addition to walking difficulties, our primary regression model identified fatigue, need for help in daily activities, and fluctuations as additional but relatively minor factors associated with FOF. Although the contributions were small (≤2.3% for each of the variables), this is, as far as we know, the first study showing that fatigue and motor fluctuations may be associated with FOF in PD. These results support the value of an interdisciplinary approach in the management of FOF including, for example, an optimization of anti-PD medications and efforts targeting independence in activities of daily living. 

There are some methodological concerns associated with this study. All data were self-reported, and future studies should consider including also clinical tests and assessments in order to provide a more complete and detailed picture. For example, “having balance problems when standing or walking” (item 6, Walk-12G) is a coarse indicator of a very complex issue. This item does not take into account the complex interaction between the person, environment and the activity at hand, and it cannot separate balance problems in standing from those connected with walking. Although this study considered a relatively broad variety of aspects, we acknowledge that there may be additional aspects influencing FOF in PD (e.g., cognitive problems, executive dysfunctions, and environmental factors). In addition, our sample was relatively limited and drawn from a university clinic. It is unknown to what extent such a sample is representative for the PD population at large, which may influence the external validity of our findings. Finally, the response rate of 68% may potentially have introduced a bias, particularly since the study design did not allow for a thorough analysis of responders versus nonresponders. However, excluded responders did not differ from those included with respect to sex, age, and PD duration, and the prevalence of FOF found here (close to 50%) is in agreement with that reported in other studies [2, 12–16]. Nevertheless, in order to gain a deeper understanding and reach firmer conclusions, additional quantitative and qualitative work is needed within this area.

## 5. Conclusions

This is to our knowledge the first study using multivariate analysis to explore factors associated with FOF in people with PD. The present results suggest that walking ability is the primary target in order to reduce FOF. Specifically, balance, climbing stairs, and turning seem to be of particular importance. Additional studies are warranted in order to further improve our understanding of FOF and how to best approach it in rehabilitation.

## Figures and Tables

**Table 1:** Sample characteristics and bivariate associations with FES(S) scores (*n* = 154).

	Total sample		Spearman correlations with FES(S) scores		*P* value
Mean (SD) age, years	70 (9.1)		−0.24		0.003
Mean (SD) PD duration, years	6 (5.4)		−0.42		<0.001
Fatigue (FACIT-F), median (q1–q3)	36 (27–42)		0.67		<0.001
Physical function (PF), median (q1–q3)	65 (40–84)		0.79		<0.001
Pain (NHP), median (q1–q3)	0 (0–25)		−0.50		<0.001
Walk-12G, median (q1–q3)	13 (6–23)		−0.82		<0.001

	*n*/total	*%*	Median (q1–q3) FES(S) scores		*P*-value^a^

*Dichotomous variables*			No	Yes	

Education: university degree	37/153	24	115 (69–130)	112 (70–130)	0.941
Living alone	38/150	25	119 (80–130)	96 (55–130)	0.125
Comorbidity	77/142	50	107 (60–130)	120 (74–130)	0.271
Motor fluctuations	90/152	58	124 (86–130)	104 (64–128)	0.010
Dyskinesia	57/153	37	124 (85–130)	93 (53–117)	<0.001
Freezing of gait^b^	57/152	37	128 (112–130)	69 (47–101)	<0.001
Turning hesitations^c^	58/150	38	128 (113–130)	69 (48–102)	<0.001
Experienced falls	50/149	33	123 (90–130)	81 (44–113)	<0.001
Experienced near falls	69/147	45	129 (111–130)	84 (52–115)	<0.001
Needing help from others in daily activities	42/153	27	124 (104–130)	59 (35–91)	<0.001
Sex, women	62/152	41	Women 116 (52–130)	Men 112 (77–130)	0.407

Possible score ranges: FACIT-F, 0–52 (higher = better); PF, 0–100 (higher = better); NHP-Pain, 0–100 (higher = worse); Walk-12G, 0–42 (higher = worse); FES(S), 0–130 (higher = better).

^a^Mann Whitney *U*-test.

^b^As assessed by item 3 (“freezing”) of the FOGQsa (Freezing of Gait Questionnaire, self-administered version). Those scoring ≥1 were categorized as freezers.

^c^As assessed by item 6 (“turning hesitations”) of the FOGQsa. Those scoring ≥1 were categorized as having turning hesitations.

FACIT-F: the Functional Assessment of Chronic Illness Therapy-Fatigue scale; FES(S): the Swedish version of the Falls Efficacy Scale; NHP: the Nottingham Health Profile; PD: Parkinson's disease; SD: standard deviation; q1–q3: first and third quartiles.

**Table 2:** Multiple linear regression with fear of falling (FES(S) scores) as the dependent variable among people with Parkinson's disease^a, b^.

Significant independent variables^c^	B (95% CI)	*β*	*P*-value	Adjusted *R* ^2^	
				Stepwise change	Cumulative
Walking difficulties (Walk 12-G)	1.7 (1.2, 2.2)	0.55	<0.001	0.680	0.680
Fatigue (FACIT-F)	0.74 (0.26, 1.2)	0.22	0.003	0.023	0.703
Turning hesitations (item 6, FOGQsa)	11 (2.5, 19.6)	0.15	0.012	0.014	0.717
Need help from others in daily activities	10 (0.96, 19)	0.13	0.030	0.010	0.727
Fluctuations	−7.6 (−15, −0.48)	−0.11	0.037	0.008	0.735

^a^For the regression analysis, scores were adjusted to be in the same direction: higher scores = more problems.

^b^Independent variables in the analysis were fatigue (FACIT-F), age (years), PD-duration (years), pain (NHP), turning hesitations (item 6, FOGQsa: dichotomized, 1 = turning hesitations), fluctuations (1 = yes), dyskinesia (1 = yes), freezing (item 3, FOGQsa: dichotomized, 1 = freezing), falls (1 = yes), near falls (1 = yes), need help from others in daily activities (1 = yes), and walking difficulties (Walk12-G).

^c^Listed by order of entry into the model (forward method).

B: regression coefficient; CI: confidence interval; *β*: standardized regression coefficient.

**Table 3:** Explorative multiple linear regression with fear of falling (FES(S) scores) as the dependent variable among people with Parkinson's disease^a, b^.

Adjusted *R* ^2^: 0.59	B (95% CI)	*β*	*P*-value
Independent variables			
Balance problems (item 6, Walk-12G)			
Not at all	Reference category		
A little	3.6 (−6.3, 13)	0.05	0.474
Moderately-extremely	26 (14, 38)	0.36	<0.001

Limited ability to climb stairs (item 5, Walk-12G)			
Not at all	Reference category		
A little	6.8 (−3.4, 17)	0.084	0.188
Moderately-extremely	27 (16, 37)	0.37	<0.001

Turning hesitations (item 6, FOGQsa)	21 (12, 30)	0.29	<0.001

^a^For the regression analysis, FES(S) scores (range, 0–130) were reversed (0 = better).

^b^Independent variables (method: enter with manual backward deletion) were: “Have you been limited in your ability to climb up and down stairs?" (item 5, Walk-12G), “Have you had problems balancing when standing or walking?" (item 6, Walk-12G), “Have you been limited in how far you are able to walk?" (item 11, Walk-12G), turning hesitations (item 6, FOGQsa), and “Has your walking been slow?" (item 12, Walk-12G).

The original five response categories of Walk-12G were recoded before being entered in the model: “not at all,” “a little,” or “moderately-quite a bit-extremely.”

B, regression coefficient; CI, confidence interval; *β*, standardized regression coefficient.
